# Genetic Markers for Tracing Introgression of Farmed Atlantic Salmon (*Salmo salar*) in Wild Conspecifics

**DOI:** 10.1111/1755-0998.70065

**Published:** 2025-10-24

**Authors:** Ingerid Julie Hagen, Kjetil Hindar, Geir Hysing Bolstad, Yann Czorlich, Ola H. Diserud, Bjørn Florø‐Larsen, Davíð Gíslason, Kevin Glover, Leó Alexander Guðmundsson, Celeste Jacq, Guðbjörg Ólafsdóttir, Stig William Omholt, Monica Favnebøe Solberg, Sæmundur Sveinsson, Harald Sægrov, Kurt Urdal, Sten Karlsson

**Affiliations:** ^1^ Norwegian Institute for Nature Research (NINA) Trondheim Norway; ^2^ Norwegian Veterinary Institute Trondheim Norway; ^3^ Matis Reykjavík Iceland; ^4^ Norwegian Institute of Marine Research Bergen Norway; ^5^ Icelandic Marine and Freshwater Research Institute Hafnarfjörður Iceland; ^6^ Nofima AS Tromsø Norway; ^7^ Blue Analytics Kong Christian Frederiks Plass 3 Bergen Norway; ^8^ Norwegian University of Science and Technology Trondheim Norway; ^9^ Rådgivende Biologer AS Bergen Norway

**Keywords:** Atlantic salmon, farmed escapees, Iceland, introgression, Norway, *Salmo salar*

## Abstract

Genetic introgression of domesticated plants and animals into wild populations occurs globally. Such introgression disrupts adaptive potential, reduces fitness in wild populations and threatens intraspecific genetic variation. The best‐documented case of farmed introgression into wild populations is that of the Atlantic salmon ( *Salmo salar* ). Norway is the world's largest producer of farmed Atlantic salmon, and the industry is growing in Iceland and other countries. In Norway, genetic introgression resulting from farmed escapees breeding with wild conspecifics has been documented in approximately two‐thirds of 250 salmon populations studied. This comprehensive quantification has been possible due to a panel of genetic markers diagnostic of farmed introgression. Improved genomic resources, continued selection and genetic drift in the farmed breeding lines, as well as new breeding lines in commercial production, call for an updated tool to quantify farmed genetic introgression. Here, we present second‐generation panels of genetic markers diagnostic of farmed introgression in Norway and the first panels of genetic markers diagnostic of farmed introgression in Iceland. We show that these diagnostic markers provide increased power to detect introgression compared to the first‐generation panel, as well as increased power compared to a genome‐wide marker set. Improved accuracy will benefit the ongoing monitoring of farmed introgression and facilitate research into the ecological and functional effects of farmed introgression in wild populations.

## 1 Introduction

Introgression of domesticated plants and animals into populations of wild conspecifics occurs globally and affects a wide range of species (e.g., maize: Hufford et al. 2013; wheat: Ellstrand et al. 2002; bison: Halbert and Derr 2007; wolves: Verardi et al. 2006). The best‐documented case is the introgression of farmed conspecifics into wild populations of Atlantic salmon ( *Salmo salar* ) (Glover et al. 2013; Karlsson et al. 2016; Wringe et al. 2018; Diserud et al. 2023). The Atlantic salmon is distributed on both sides of the North Atlantic Ocean and is separated into different phylogenetic groups (Bourret et al. 2013). Within each phylogenetic group, the species has a metapopulation structure that consists of numerous genetically distinct populations (Bourret et al. 2013). The Atlantic salmon is listed as Near Threatened by the international IUCN Red List (Sayer 2024), with a declining trend and many populations being extinct. The largest wild resource of Atlantic salmon is found in Norway (ICES 2019), where two phylogenetic groups are represented: the East Atlantic group and the Barents/White Sea group (Wennevik et al. 2019; Bourret et al. 2013). Norway is also the world's largest producer of farmed Atlantic salmon, with a recent production of 1.5 million tons per year and individuals in net pens (c. 450 million) outnumbering wild conspecifics 1000‐fold (Ahlbeck‐Bergendahl and April 2019). Norwegian breeding lines were founded from wild salmon of the East Atlantic phylogenetic group (Gjedrem et al. 1991; Gjøen and Bentsen 1997), and domesticated salmon is therefore more divergent from the Barents/White Sea phylogenetic group than from the East Atlantic group (Glover et al. 2013; Karlsson et al. 2016). While salmon farming has been ongoing in Norway since the late 1960s, the history of the industry in Iceland is much shorter and currently on a much smaller scale. Yet, production in Iceland has sharply increased over the past decade, reaching around 45,000 tons per year, and is expected to grow further. The commercial breeding line used in net pens in Iceland since 2000—Stofnfiskur—is originally derived from an admixture of several Norwegian farmed lines (Karlsson et al. 2017). The wild Atlantic salmon population in Iceland is approximately one‐tenth the size of that in Norway (ICES 2025). It consists of two phylogenetic groups (here denoted North and South) that are different from those in Norway (Bourret et al. 2013; Olafsson et al. 2014) and exhibits greater genetic divergence from farmed conspecifics in Iceland than the wild populations in Norway do.

Each year, thousands to hundreds of thousands of farmed salmon escape from net pens along the Norwegian coast (Skilbrei et al. 2015). In Iceland, farmed escapees are observed in many rivers, and significant farmed genetic introgression in wild populations has been documented (Guðmundsson et al. 2023). Whilst survival of escapees is generally low (Hansen 2006; Skilbrei et al. 2015), some find their way to the spawning grounds of native populations where they in some years may outnumber adult wild salmon (Sægrov et al. 1997; Fiske et al. 2006), thus enabling gene flow of farmed genotypes into wild populations despite the overall low reproductive success of escaped farmed salmon (Fleming et al. 1996, 2000).

Due to 14 generations of directional selection for economically important production traits (Gjedrem et al. 1991; Gjøen and Bentsen 1997), as well as genetic drift in breeding lines and relaxed natural selection, farmed salmon now differ from wild salmon both genetically and phenotypically (Glover et al. 2017). Theoretical modelling predicts that the fitness consequences of domesticated introgression in wild populations will be negative (Tufto 2001, 2010; Castellani et al. 2018). All available evidence on the effects of introgression of farmed Atlantic salmon in wild populations supports this prediction. The altered phenotype of farmed salmon is not congruent with the selective optimum in the wild, and consequently, farmed salmon have lower fitness and survival in the natural environment compared to wild conspecifics (Fleming et al. 2000; McGinnity et al. 1997; Skaala et al. 2012, 2019). Hybrid offspring of farmed and wild salmon display intermediate phenotypes (Solberg et al. 2013; Skaala et al. 2019) and lower fitness compared to wild individuals (McGinnity et al. 2003; Wringe et al. 2018; Skaala et al. 2019) whilst the presence of farmed and hybrid juveniles in rivers may negatively impact the survival of wild juveniles (Sundt‐Hansen et al. 2015; Robertsen et al. 2019) through resource competition (Skaala et al. 2012, 2019). Farmed genetic introgression can therefore decrease the viability of wild populations (Hindar et al. 2006; Skaala et al. 2019; Wacker et al. 2021) and genetic introgression of escaped farmed Atlantic salmon is considered one of the most significant challenges to environmentally sustainable aquaculture (Taranger et al. 2015) and to wild salmon populations in Norway (Forseth et al. 2017).

To quantify the extent of farmed genetic introgression in Atlantic salmon, a ‘first‐generation’ panel of Single Nucleotide Polymorphism (SNP) markers that differentiate between Norwegian wild and farmed salmon independent of population origin was identified (Karlsson et al. 2011). This first‐generation panel was derived from 4514 genome‐wide SNPs genotyped for three major breeding lines in commercial production and historical samples from 13 Norwegian wild populations (Karlsson et al. 2011). Using a subset of these markers, a standardised method to quantify unidirectional genetic introgression was developed (Karlsson et al. 2014). Applying this approach, introgression has been quantified in 250 salmon populations, which together represent more than 90% of the Norwegian wild salmon resource (Glover et al. 2013; Karlsson et al. 2016; Diserud et al. 2023). This extensive characterisation of the genetic integrity of wild populations shows that introgression of farmed salmon in wild populations in Norway is widespread and that only one‐third of wild populations show no sign of farmed introgression. Similar tools have also been developed for Canadian populations of Atlantic salmon (Wringe et al. 2018) and are under development for Scottish populations (Gilbey et al. 2021).

The first‐generation marker panel has allowed farmed genetic introgression in wild salmon populations to be documented and quantified at an extent that surpasses similar information in any other species, but an updated version of this tool is necessary for several reasons. New high‐density SNP‐arrays that capture genome‐wide linkage disequilibrium have been developed, with subsequently greater chances of detecting SNPs that differentiate between wild and farmed salmon. Karlsson et al. (2011) did not differentiate between the Norwegian phylogenetic groups, and although the first‐generation marker panel has proven useful for quantifying farmed genetic introgression also for individuals and populations belonging to the Barents/White Sea group (Bolstad et al. 2017; Diserud et al. 2019), a panel specific to this phylogenetic group is required. Also, there is a need for increased accuracy in quantifying farmed genetic introgression on the individual level, which will facilitate research into the functional effects of farmed introgression in wild individuals (Bolstad et al. 2017, 2021; Besnier et al. 2022). Moreover, the genetic composition of salmon in commercial production has changed since Karlsson et al. (2011) due to genetic drift and selection within the breeding nuclei, and there is also increased use of two additional breeding lines: Rauma and Stofnfiskur. In Iceland, a standardised tool to quantify farmed salmon introgression into wild populations has yet to be established, and the identification of markers diagnostic of farmed ancestry is a first step towards a tool that can be applied to monitor the genetic integrity of wild populations. Using recent samples from all breeding nuclei used in Norway and Iceland together with samples from wild populations, we present panels of markers diagnostic of farmed introgression that are designed for each of the phylogenetic groups in Norway and Iceland. We show how the new marker panels for Norwegian salmon provide improved accuracy in detecting farmed introgression compared to the first‐generation panel and that the selection of diagnostic SNPs provides higher accuracy than a genome‐wide marker panel.

## 2 Material and Methods

### 2.1 Genotyping

**FIGURE 1:**
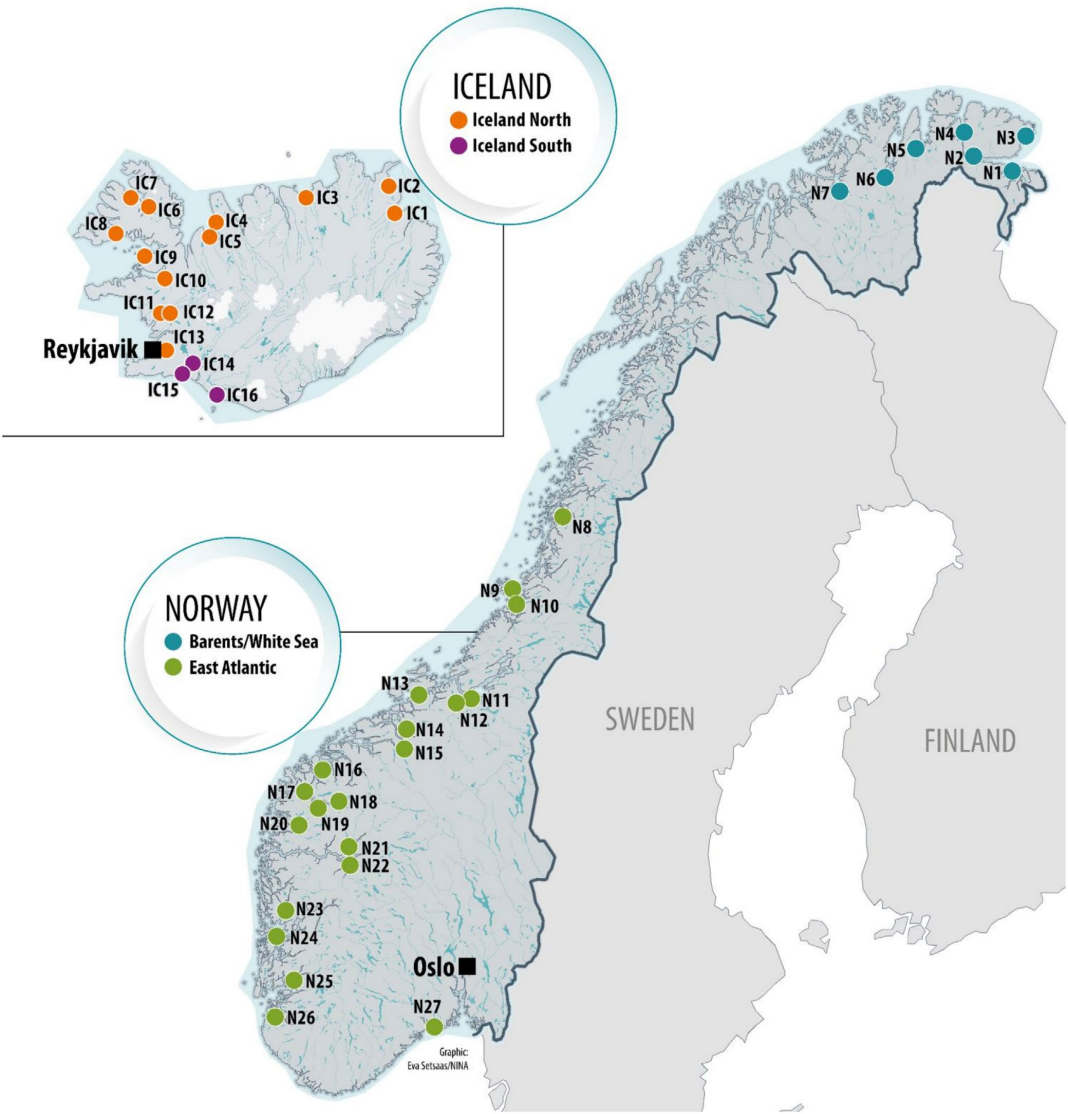
Maps of Norway and Iceland illustrating the locations of the estuaries for each of the rivers from which Atlantic salmon were collected. The different colours represent different phylogenetic groups.

The following individuals were genotyped on Aquagen's 60 K Axiom SSATRACK array: (i) 721 individuals representing the five breeding lines currently used in Norway (Aquagen, Salmobreed, Mowi, Rauma and Stofnfiskur), of which the latter is the only breeding line used in Iceland, (ii) 1029 individuals from 27 wild populations covering the entire Norwegian coast and both phylogenetic groups, and 369 wild Icelandic individuals from 16 populations, also representing the geographic distribution of salmon populations in Iceland and the two main phylogenetic groups present (Figure 1, Figure S1). See Table S1 for information about the number of individuals per population and breeding nuclei year classes. Twenty individuals of varying DNA quality were genotyped twice to assess genotype consistency across markers. Because a poor genotype rate within individuals was associated with a higher error rate for the remaining genotypes, samples with more than 3.1% missing genotypes were removed. We also removed markers with more than 10% error observed in 20 duplicated pairs and a minor allele frequency below 0.05, leaving 57,545 markers to be included in downstream analyses. Populations in the hybrid zone between the East Atlantic and Barents/White Sea phylogenetic groups in Norway were avoided so that the marker panels would be specifically adapted to each group. Both recent (2016–2018) and older (1980s–1990s) Norwegian samples were initially genotyped, but poor call rates caused most of the older samples to be removed from the final data. From Iceland, only recent samples (2015–2018) were genotyped. All the Norwegian samples were classified as being of wild ancestry derived from a *P*(wild) value > 0.71 according to the first‐generation marker panel and corresponding baseline references to quantify farmed genetic introgression (Karlsson et al. 2014). In Iceland, no such systematic screening was done; instead, all available information was used to include only individuals that putatively were unaffected by farmed introgression. For example, all individuals from populations IC6–IC9 were previously genotyped using microsatellites, and based on this marker set, the ancestry of these individuals was inferred to be wild.

### 2.2 Identifying SNPs That Are Consistently Divergent Between Breeding Nuclei and Wild Populations

Norwegian wild populations are divided into the Barents/White Sea and East Atlantic phylogenetic groups, whilst the Icelandic samples are divided into phylogenetic groups denoted North and South (Figure 1). Separate analyses were conducted for each of the four phylogenetic groups: First, allele frequencies for each SNP were calculated for each of the breeding nuclei and the wild phylogenetic group, where the latter was treated as a single group (not considering population). Second, we calculated the fixation index (*F*_ST_) for each SNP using the method developed by Weir and Cockerham (1984), by comparing the pooled wild group to the pooled farm population (treating the farm individuals as one population regardless of breeding line). Allele frequency calculations and *F*_ST_ estimates were carried out using VCFtools (Danecek et al. 2011). Next, we selected the SNPs for which the allele frequency in the breeding nuclei was consistently higher or lower than the frequency observed in the wild group. Thus, the observed frequency for a given allele in each of the five breeding lines had to be above or below the one observed in each of the wild phylogenetic groups. This produced a list of SNPs for which the allele frequency difference between the five breeding nuclei and the wild group had to be all negative or all positive. Next, we produced a subset of SNPs for which the minimum observed difference in allele frequency and pairwise difference in *F*_ST_ between any farm line and the wild population was over 0.05 for both parameters.

### 2.3 Identifying SNPs Under Natural Selection

For tracing farmed ancestry in wild populations, it is important that estimates of introgression are not biased by natural selection acting on specific SNPs. Markers under strong natural selection in the wild were identified using the *F*_ST_‐Heterozygosity Outlier approach (Flanagan and Jones 2018) implemented in the fsthet package (Flanagan and Jones 2018), which runs in the statistical software environment R (R Core Team 2021). The fixation index (*F*_ST_), representing the genetic differentiation among populations, and the expected heterozygosity were calculated separately for each phylogenetic group using the R package outflank (Whitlock and Lotterhos 2015). Loci were categorised into 40 equal‐sized bins based on their expected heterozygosity. Bins with fewer than 20 loci were merged with neighbouring bins. Loci were sorted by *F*_ST_ values within each bin, and quantiles were calculated. Markers with *F*_ST_ values lying outside the 0.95 quantiles were identified as outliers. The fsthet method is based on the empirical *F*_ST_ ‐heterozygosity values and does not assume a null population genetics model (Flanagan and Jones 2018).

### 2.4 Selecting SNPs for the Four Panels

The candidate markers described above were filtered to remove markers identified as being under strong natural selection. We also removed markers within 100,000 bp of those found to be under selection, thus accounting for linkage disequilibrium (LD) with markers under selection. The threshold of 100,000 bp was chosen as LD in wild populations is weak at this level of distance (Kijas et al. 2017), and most LD is lost in the farmed populations (Barria et al. 2018). Moreover, to increase the probability of even segregation of the markers during meiosis, we selected markers not closer than approximately 5 cM, based on chromosome‐wise average recombination rate on the sex‐averaged linkage map (Lien et al. 2011). Next, we selected the markers with the highest pairwise *F*_ST_ between the pooled farmed group (the five breeding lines) and the pooled wild group (populations within each phylogenetic group), starting with the marker with the highest *F*_ST_ on each of the 29 chromosomes, out of which the 25 ‘best’ markers were selected. Next, another 25 markers were added, again on different chromosomes such that all chromosomes gained at least one of the ‘best’ 50 diagnostic SNP markers and were spaced widely on the chromosomes to ensure even segregation during meiosis. We cumulatively added markers evenly spaced on the chromosomes to obtain marker sets of 25, 50, 75, 100, 125, 150, 200 and 250 markers, respectively. Due to the criteria of at least 5 cM distance between the markers and areas with many markers with high *F*_ST_ close together, 250 was the upper limit of markers that could be fitted.

### 2.5 Estimating Wild Ancestry—*P*(Wild)

We evaluated the power of the selected SNPs to differentiate wild and farmed salmon by estimating the probability of belonging to wild (*P*(wild)) versus farmed salmon, as outlined by (Karlsson et al. 2014). *In silico* generated center‐point populations were generated for the farmed salmon and for each phylogenetic group, respectively. Equal sample sizes from each of the wild populations were pooled and a population was created based on random mating using Hybridlab (Nielsen et al. 2006). Processing one individual at a time, each wild and farmed salmon was analysed with 100 individuals of the *in silico* generated center‐point populations of wild and farmed salmon in STRUCTURE (Pritchard et al. 2000) by assuming two populations (*K* = 2), with 50,000 repetitions as burn‐in and 100,000 repetitions after burn‐in, and without a priori information of origin. The estimated probability of belonging to wild salmon (*P*(wild)) was extracted from the output files and the power of differentiating between wild and farmed salmon was explored from the probability distributions. We estimated *P*(wild) using the top 25, 50, 75, 100, 125, 150, 200 and 250 SNPs. For evaluating the improvement of the new SNP panels compared to the SNPs identified by Karlsson et al. (2011), we applied the 48 SNPs that have routinely been used for monitoring farmed genetic introgression in Norway (Karlsson et al. 2016; Diserud et al. 2023).

### 2.6 Simulation of F1‐Hybrids

For each phylogenetic group, we started with one wild salmon population representing this phylogenetic group, and samples from all the breeding lines relevant for this region. The N7 population was used for the Barents/White Sea group, and N10 was used for the East Atlantic group. For Iceland North, we used IC9, and for Iceland South, we used IC15. As potential generation 0 parents, 1000 individuals were *in silico* generated from each population (Wild and Farmed), each with 250 SNPs, using HybridLab (Nielsen et al. 2006). Next, we used the statistical software environment R to simulate F1‐hybrids by first sampling randomly (with replacement) one spawner from the wild and one from the farmed population. For this pair of spawners, we made an offspring by generating a gamete from each parent, sampling randomly an allele from each SNP, and then merging these assuming no linkage. This step was then repeated 1000 times, simulating F1‐hybrids with different pairs of parents from each combination of wild population × farmed breeding line. For each phylogenetic group, F1‐hybrids were simulated between the wild salmon population and all relevant farmed breeding nuclei.

## 3 Results

**FIGURE 2:**
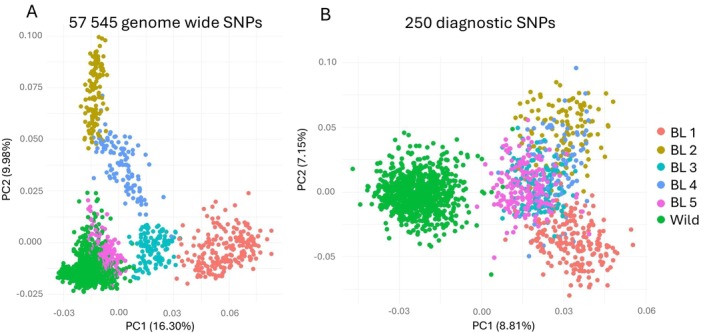
Principal Component Analysis using 57,545 genome wide SNPs (A) and the 250 diagnostic SNPs (B) illustrated with wild populations from the East Atlantic phylogenetic group (Wild) and samples from the five farmed breeding lines (BL) that are used in Norway.

Pairwise *F*_ST_‐values between the pooled farmed group and the four wild groups were higher when using the 250 diagnostic SNPs, compared to the genome‐wide marker panel with 57,545 SNPs (Table 1). The increased *F*_ST_‐values when using the 250 diagnostic markers illustrate the increased separation gained from selecting a subset of the most divergent markers. For the East Atlantic phylogenetic group, the wild and farmed clusters partly overlap when using the initial 57,545 SNPs (Figure 2A). Applying the 250 most diagnostic SNPs increases the distance between the wild cluster and the farmed cluster (Figure 2B), as expected according to the increased *F*_ST_ between the wild group and the different farmed populations. The most important improvement is the decrease in genetic distances within the farmed group. *F*_ST_‐values when using the 48 SNPs in the first‐generation marker panel (Karlsson et al. 2011) were 0.055 for farmed versus East Atlantic and 0.132 for farmed versus the Barents/White Sea group (Table 1). Thus, with the samples used in this study, *F*_ST_‐values between wild and farmed groups have nearly doubled for the East Atlantic group (to 0.094) and more than doubled for the Barents/White Sea group (to 0.322, Table 1) when applying the second‐generation diagnostic SNPs. The four ranked lists (one list for each phylogenetic group) of diagnostic SNP markers and their respective *F*_ST_ values and genomic positions are in Table S2.

**TABLE 1:** Pairwise *F*_ST_ values for farmed salmon versus four different phylogenetic groups of wild salmon.

Wild group	57,545 SNPs	250 diagnostic SNPs	1.gen. 48 SNPs
East Atlantic	0.014	0.094	0.055
Barents/White Sea	0.051	0.322	0.132
Iceland North	0.120	0.579	—
Iceland South	0.118	0.570	—

*Note:* The *F*_ST_ values were calculated for the genome‐wide tool with 57,545 SNPs, the 250 diagnostic SNPs identified, and the first‐generation panel of 48 SNPs.

Because SNPs that differentiated substantially between the respective wild populations within each phylogenetic group were removed (to avoid selecting SNPs under strong natural selection), and because we selected SNPs with a generic level of allele frequency among wild populations that were different from a generic level of allele frequency among the breeding nuclei, global *F*_ST_ within the wild groups was lower when the 250 diagnostic SNPs were used, compared to when global *F*_ST_ was derived from 57,545 genome‐wide SNPs or from a random subset of 250 SNPs (Table 2). This decrease in global *F*_ST_ is expected and means that while differences between farmed and wild individuals are accentuated, differences between natural populations are masked. However, this decrease in global *F*_ST_ did not apply to the wild populations in the East Atlantic group. This is explained by the small differences between populations in the East Atlantic group compared to the relatively large differences between the breeding nuclei.

**TABLE 2:** Global *F*_ST_ for wild populations within each phylogenetic group derived from 57,545 genome wide SNPs, 250 diagnostic SNPs and 250 SNPs that were randomly selected.

Phylogenetic group	57,545 SNPs	250 diagnostic SNPs	250 random SNPs
East Atlantic	0.016 (0.009)	0.016 (0.010)	0.016 (0.010)
Barents/White Sea	0.025 (0.008)	0.019 (0.005)	0.025 (0.010)
Iceland North	0.044 (0.024)	0.038 (0.022)	0.047 (0.030)
Iceland South	0.050 (0.040)	0.032 (0.025)	0.054 (0.047)

*Note:* Numbers in brackets are the standard deviation, which describes the variation of *F*_ST_‐values within each group.

### 3.1 Separation Between Wild and Farmed Salmon

**FIGURE 3:**
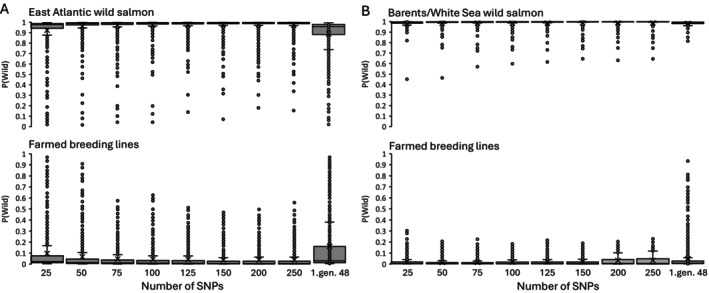
Box and whisker plot of *P*(wild) estimates for wild salmon in the East Atlantic phylogenetic group and farmed salmon (panel A) and wild salmon in the Barent/White Sea phylogenetic group and farmed salmon (panel B) using different numbers of SNP‐markers selected for differentiating between wild and farmed salmon. The first‐generation 48 SNPs were previously identified by Karlsson et al. (2011). Boxes represent the first and last quartile, × indicates the means and horizontal lines within boxes are the median. The whiskers represent the minimum and maximum value that is larger or smaller than 1.5 times the interquartile range. Dots outside this range are regarded as outliers.

**FIGURE 4:**
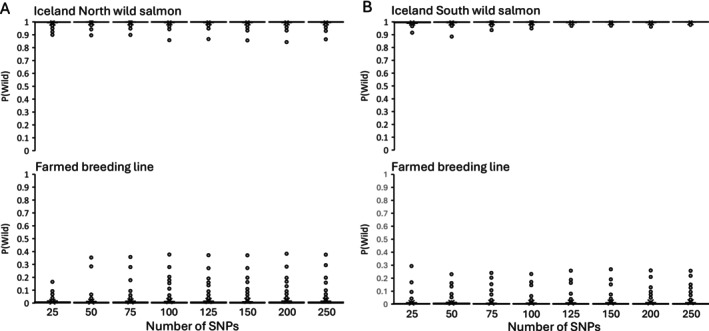
Box and whisker plot of *P*(wild) estimates for wild salmon in the Iceland North phylogenetic group and farmed salmon (panel A) and the Iceland South phylogenetic group and farmed salmon (panel B) using different numbers of SNP markers selected for differentiating between wild and farmed salmon. The farmed salmon is represented by the one breeding line used in Iceland. The components of the box and whisker plot are described in Figure 3.

The farmed breeding lines used in Norway, including Stofnfiskur, which is the only breeding line used in Iceland, originate from the East Atlantic phylogenetic group. Hence, separation between farmed and wild East Atlantic salmon is weaker (Figure 3A), compared to separation between farmed and wild salmon from the Barents/White Sea group (Figure 3B) and between farmed salmon (only Stofnfiskur) and wild salmon in Iceland (Figure 4A,B). Nevertheless, the selected markers performed well in separating wild and farmed salmon also in the East Atlantic group. The separation of wild and farmed salmon improved considerably with an increasing number of SNPs from 25 to 100 SNPs, but less from 125 to 250 SNPs (Figure 3A,B). With the 250 diagnostic SNPs the average *P*(wild) estimate was 0.07 with a 95 percentile of 0.22 for farmed salmon, and on average 0.97 with a 5 percentile of 0.86 for wild salmon. With the 48 SNPs in the first‐generation marker panel (Karlsson et al. 2011) genotyped on the individuals used in this study, the average *P*(wild) was 0.16 with a 95 percentile of 0.84 for the farmed salmon, and on average 0.89 with a 5 percentile of 0.51 for the wild salmon. There was a diagnostic separation between wild and farmed salmon in the Barents/White Sea phylogenetic group and the Icelandic phylogenetic groups with no overlap in *P*(wild) estimates, even with only 25 SNPs used (Figures 3B and 4A,B).

### 3.2 Detection of F1 Hybrids

**FIGURE 5:**
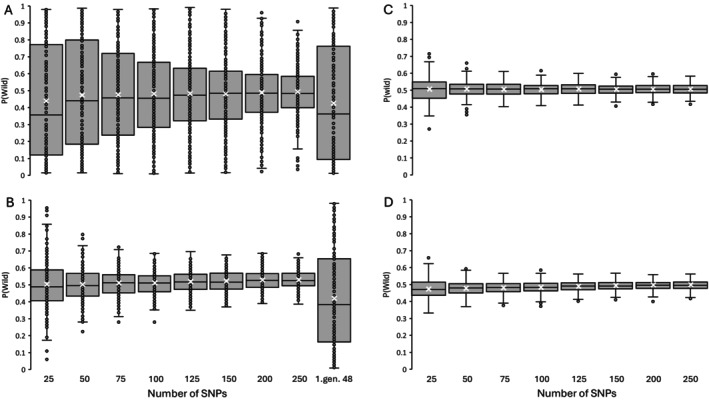
Box and whisker plot of *P*(wild) distribution of simulated F1‐hybrids between farmed salmon and a wild salmon population in (A) the East Atlantic phylogenetic group, (B) the Barents/White Sea group, (C) Iceland North and (D) Iceland South, using different numbers of selected SNPs. The first‐generation 48 SNPs were previously identified by Karlsson et al. (2011). The components of the box and whisker plot are described in Figure 3.

Analyses of *in silico* generated F1‐hybrids showed that the identification of individual hybrids is more challenging in the East Atlantic phylogenetic group (Figure 5A) than in the Barents/White Sea group (Figure 5B) and in Icelandic salmon (Figure 5C,D), where there is a much narrower distribution of *P*(wild) estimates around 0.5. In all phylogenetic groups, the identification of F1‐hybrids becomes more certain with an increasing number of SNPs used, and especially in the East Atlantic group. The new panel of 250 diagnostic markers performed much better than the 48 SNPs in the first‐generation marker panel. For East Atlantic F1‐hybrids, the average *P*(wild) was 0.49 (95% CI = [0.26, 0.74]), using 250 SNP‐markers. Corresponding average *P*(wild) was 0.53 (95% CI = [0.44, 0.62]) for the Barents/White Sea group. For the Icelandic F1‐hybrids, the *P*(wild) estimates were tightly clustered around 0.5, with an average of 0.51 (95% CI = [0.46, 0.55]) for Iceland North and an average of 0.50 (95% CI = [0.45, 0.55]) for Iceland South.

## 4 Discussion

In this study, we present genetic markers that collectively are diagnostic of farmed genetic introgression in four different phylogenetic groups of wild Atlantic salmon. We show that the diagnostic markers provide increased separation to detect farmed introgression compared to a genome‐wide marker set, particularly for the East Atlantic group from which farmed Atlantic salmon is derived. Moreover, the marker panels for the two phylogenetic groups in Norway represent improved tools to quantify farmed genetic introgression compared to the first‐generation tool (Karlsson et al. 2011, 2014), and the two panels designed for Icelandic salmon represent novel tools to quantify farmed introgression in this region. Introgression of domesticated genotypes occurs in a range of species (Ellstrand et al. 2002; Adavoudi and Pilot 2022), and while the presented marker panels are specific to Atlantic salmon, the approach we have used to identify the diagnostic SNPs and account for natural selection and large variation in the farmed group can be applied to other species.

An important reason for the increased accuracy of the marker sets presented here compared to the first‐generation marker set (Figures 3 and 5) is the dataset from which the diagnostic markers were derived. The first‐generation marker set was selected from 4514 initial markers, which is not enough to capture linkage disequilibrium along the salmon genome (Kijas et al. 2017), and many genomic regions with high *F*_ST_ between the farmed groups and the wild groups would have been undetected. The new diagnostic markers were selected from 57,545 SNPs, and the mean distance between markers using this large dataset was around 39,000 base pairs, which captures most LD along the genome (Kijas et al. 2017), and we were therefore able to detect nearly all genomic regions where the wild and farmed groups differed significantly. The use of recent samples to represent the breeding nuclei will also have contributed to the improved accuracy of the second‐generation panels for Norwegian salmon. Farmed salmon is continuously selected for commercial traits (Gjedrem and Baranski 2010), and the relatively small effective population size in the breeding nuclei (Karlsson et al. 2010) leads to strong genetic drift in the different farmed populations. Around 3–5 salmon generations have passed from the farmed baseline in the first‐generation panel (year class 2000) to the second‐generation panels (year classes 2012–2017). This means that the genetic distance between wild and farmed populations will likely have increased, and we expect that the new marker panels provide a better representation of the contemporary farmed populations than the first‐generation panel.

Selection and genetic drift in the farmed breeding nuclei over 14 generations (Gjedrem et al. 1991; Gjøen and Bentsen 1997) have caused substantial differentiation also between the different farmed populations (Figure 2A). Such large genetic variation in the farmed group and close relatedness to the East Atlantic group from which the farmed group was derived represent a challenge when selecting markers that differentiate between the two groups. The Barents/White Sea group and Icelandic salmon are more genetically different from the farmed group, which facilitates the selection of diagnostic SNPs, despite the large genetic variation in the combined farmed group. For the East Atlantic group, the detection of second‐generation hybrids (usually backcrossed to wild) may still be challenging but will likely be possible in the Barents/White Sea group and can be done with high accuracy in Icelandic salmon (Guðmundsson et al. 2023). Because aquaculture and escaped farmed salmon have been present for a long time in Norway (Diserud et al. 2019), with many generations of introgression in wild populations, there are potentially many hybrid and backcrossed classes of different breeding line origins, with overlapping distributions of estimated introgression. Therefore, determining an individual's specific hybrid class will be difficult, especially after several generations of introgression.

The large genetic divergence between the Norwegian phylogenetic groups and the Icelandic phylogenetic groups (Figure S2) would not have been possible if gene flow from wild Norwegian salmon to Icelandic populations was common. In the unlikely event that wild Norwegian strays are encountered in Icelandic rivers, such individuals will probably be diagnosed as farmed escapees when applying the Icelandic genetic marker panels presented here. However, in cases where such individuals are suspected to be of wild Norwegian origin, the second‐generation Norwegian marker panels can be used to verify the genetic origin.

SNPs likely involved in local adaptation in wild populations may not accurately reflect the true farmed ancestry after generations of introgression and contemporary selection against specific genotypes, and SNPs under strong natural selection were therefore removed from the panel of candidate SNPs. This approach will increase the accuracy in characterising farmed genetic introgression in wild populations but also removes some markers that are informative of genetic differences between wild populations, as illustrated by the lower global *F*_ST_ within each phylogenetic group when the 250 diagnostic SNPs are used (Table 2).

All wild Norwegian samples used in this study were previously genotyped on the first‐generation panel and with the old wild and farmed baselines (Karlsson et al. 2014) to quantify farmed genetic introgression. Only individuals with a low probability of farmed ancestry were included in the wild baselines in the present study. Because the first‐generation panel lacked accuracy on the individual level (Karlsson et al. 2014), it is possible that some individuals with partial farmed ancestry were included in wild baselines. In Iceland, efforts were made to use only wild individuals unaffected by farmed introgression, but also here, some introgressed individuals may have been included in the baselines. As the approach for selecting diagnostic markers was based on allele frequency differences between the wild and the farmed groups, a low number of introgressed individuals will not have shifted the allele frequencies substantially, and the inclusion of some introgressed individuals in the baselines will therefore have minimal impact on the outcome. The potential presence of introgressed individuals in the wild baselines may explain some of the outliers observed in the *P*(wild) distributions (Figures 3 and 4).

Applying the first‐generation 48 SNP‐panel with the baselines presented here generated many low and high *P*(wild) values in wild and farmed salmon, respectively, despite wild individuals being initially selected to have high *P*(wild) values. The main reason for this is the introduction of two new breeding lines (Stofnfiskur and Rauma) in the farmed baselines. Because individuals were initially selected based on the first‐generation 48 SNPs, we expect that the *P*(wild) distributions with these SNPs show a better separation between wild and farmed salmon than they would have done without the prior selection of individuals. Hence, the improvement of the new panel of SNPs in relation to the first‐generation 48 SNP panel is probably better than suggested by the results presented here.

When the new panels are implemented to trace farmed genetic introgression, it will be necessary to genotype individuals collected from populations before aquaculture was introduced to the Norwegian coast in order to create wild salmon baselines for each phylogenetic group. For populations in the phylogenetic hybrid zone (Wennevik et al. 2019), baselines specific to these will be generated. In cases where DNA samples of historical individuals are not available (for instance in many rivers in Iceland), we suggest that modern samples are analysed, and that individuals identified as hybrids are removed from the wild baseline. Identification of first‐generation hybrids and backcrosses to wild salmon should be possible with high accuracy for Icelandic salmon and probably also in the Barents/White Sea phylogenetic group using the new diagnostic markers. Genotyping of historical samples with somewhat degraded DNA can be difficult on hybridization‐based SNP arrays, such as the 60 K array used here. PCR‐based techniques, on the other hand, can successfully genotype samples of poor DNA quality (Karlsson et al. 2016). Genotyping a subset of diagnostic SNPs therefore provides higher accuracy in detecting farmed introgression, allows for historic samples to be included in the wild baselines, and also has the benefit of a more rapid throughput of smaller sample volumes compared to high‐density genome‐wide genotyping. In wild populations supplemented with hatchery‐produced individuals, introgressed broodstock leads to unwanted genetic effects in the wild (Hagen et al. 2019). Rapid genotyping has been crucial in the Norwegian broodstock screening program, where individuals are genetically screened for farmed genetic introgression and rejected as broodstock if farmed ancestry is detected (Hagen and Karlsson 2023). In Iceland, no broodstock control is currently in place, and the Icelandic marker panels presented here will allow for the development of similar tools and pipelines in Icelandic stocking programs. Applications where high accuracy on the individual level, such as the broodstock control program and research into the functional effects of farmed introgression (Bolstad et al. 2017, 2021; Besnier et al. 2022), will benefit from increased accuracy on the individual level. We expect that the new marker panels will be a valuable resource for the ongoing monitoring of farmed genetic introgression in Norway and future monitoring in Iceland and to facilitate research into ecological and functional effects of farmed introgression in wild populations.

## Author Contributions

Conceived of the study: K.H., O.H.D., C.J., K.G. and S.K. Data curation: I.J.H., K.H., L.A.G. and S.K. Formal analyses: I.J.H., G.H.D., Y.C. and O.H.D. Funding acquisition: K.H., D.G., L.A.G. and S.K. Investigative analyses: I.J.H., G.H.B., Y.C., O.H.D., L.A.G. and S.K. Developed methodology: I.J.H., G.H.B., Y.C., O.H.D. and S.K. Project administration: K.H., D.G., L.A.G. and S.K. Provided resources: K.H., B.F.‐L., D.G., K.G., L.A.G., C.J., G.Ó., S.W.O., M.F.S., H.S., S.S., K.U. and S.K. Original draft: I.J.H., K.H., Y.C., O.H.D., L.A.G. and S.K. All authors contributed to the revision of the final draft. S.K. led the study and is the senior scientist.

## Disclosure

*Benefits Generated*: Benefits from this research accrue from the sharing of our data and results on public databases as described above.

## Conflicts of Interest

The authors declare no conflicts of interest.

## Supporting information


**Table S1:** Sample sizes for each wild population and each breeding nucleus. Information about year classes is included for the breeding nuclei.
**Figure S1:** Principal Component Analysis using 57 545 genome wide SNPs illustrating the genetic distance between the two phylogenetic groups in Iceland (denoted with different symbols) and the 16 wild populations (IC1‐IC16 denoted with different colorus), and how the populations belong to either Iceland North or Iceland South.
**Figure S2:** Principal Component Analysis using 57,545 genome wide SNPs illustrating the genetic distance between the different phylogenetic groups in Norway and Iceland. The *F*_ST_ between the wild Norwegian Barents/White Sea and the wild Norwegian East Atlantic phylogenetic groups is 0.042. The *F*_ST_ between the wild Norwegian East Atlantic phylogenetic group and the pooled Icelandic group is 0.112. The *F*_ST_ between the wild Norwegian Barents/White Sea phylogenetic group and the pooled Icelandic group is 0.117.

**Table S2:** The 250 diagnostic SNP markers with respective *F*_ST_ values and genomic position for each phylogenetic group. Markers are listed according to their ranking.

## Data Availability

The data that support the findings of this study are openly available in Zenodo at https://zenodo.org/records/15624314.
