# Supplementary material for: Genetic Markers for Tracing Introgression of Farmed Atlantic Salmon (Salmo salar) in Wild Conspecifics
Source: Mol Ecol Resour. 2025 Oct 24;26(1):e70065. doi: 10.1111/1755-0998.70065 (PMC12627911; doi:10.1111/1755-0998.70065)
Supplement: Supplementary file 1 — Table S1: Sample sizes for each wild population and each breeding nucleus. Information about year classes is included for the breeding nuclei. Figure S1: Principal Component Analysis using 57 545 genome wide SNPs illustrating the genetic distance between the two phylogenetic groups in Iceland (denoted with different symbols) and the 16 wild populations (IC1‐IC16 denoted with different colorus), and how the populations belong to either Iceland North or Iceland South. Figure S2: Principal Component Analysis using 57,545 genome wide SNPs illustrating the genetic distance between the different phylogenetic groups in Norway and Iceland. The F ST between the wild Norwegian Barents/White Sea and the wild Norwegian East Atlantic phylogenetic groups is 0.042. The F ST between the wild Norwegian East Atlantic phylogenetic group and the pooled Icelandic group is 0.112. The F ST between the wild Norwegian Barents/White Sea phylogenetic group and the pooled Icelandic group is 0.117. [file MEN-26-e70065-s002.docx]

**Supplementary Table 1:** Sample sizes for each wild population and each breeding nucleus. Information about year classes is included for the breeding nuclei.

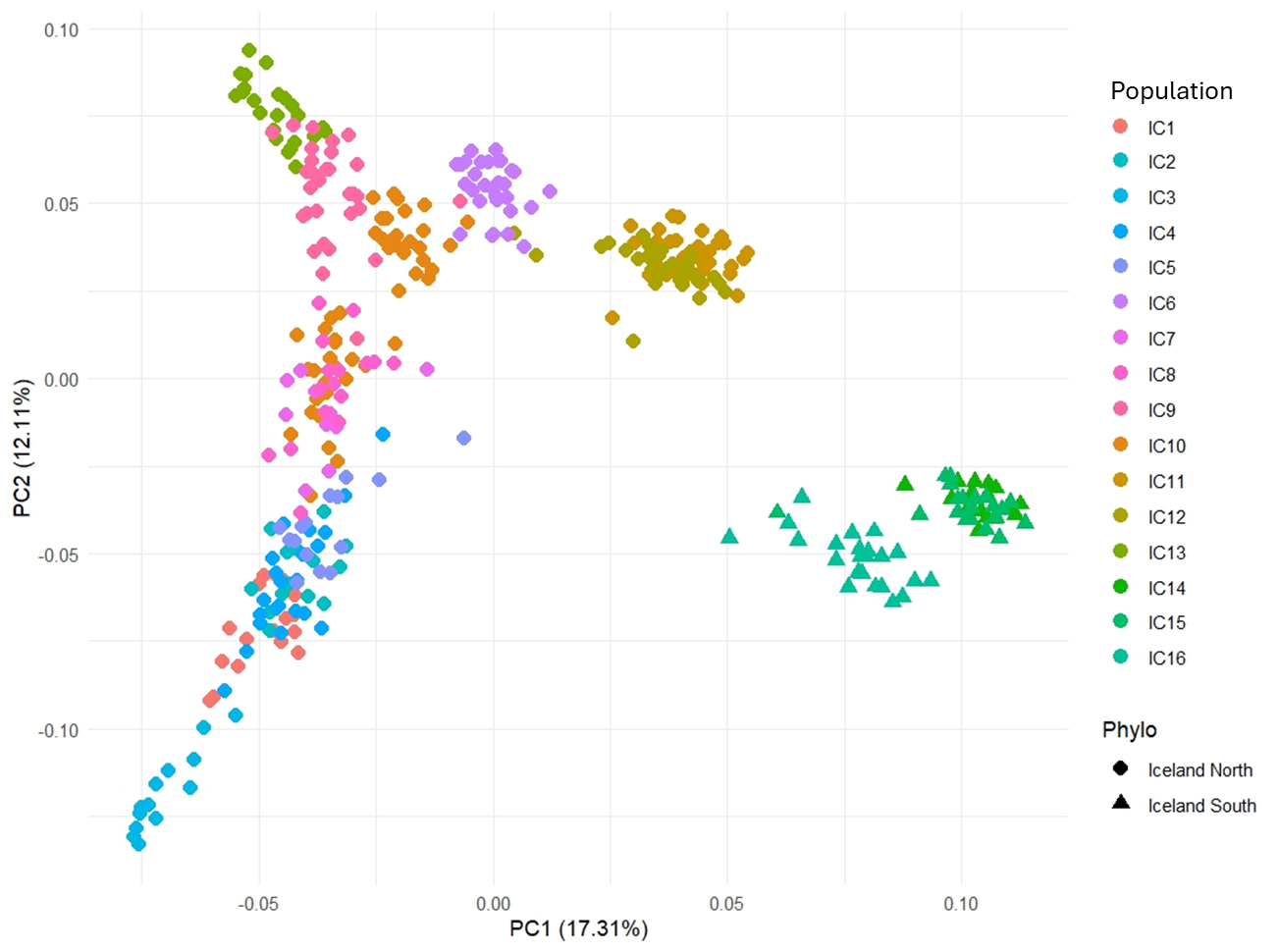


**Supplementary Figure 1:** Principal Component Analysis using 57 545 genome wide SNPs illustrating the genetic distance between the two phylogenetic groups in Iceland (denoted with different symbols) and the 16 wild populations (IC1-IC16 denoted with different colors), and how the populations belong to either Iceland North or Iceland South.


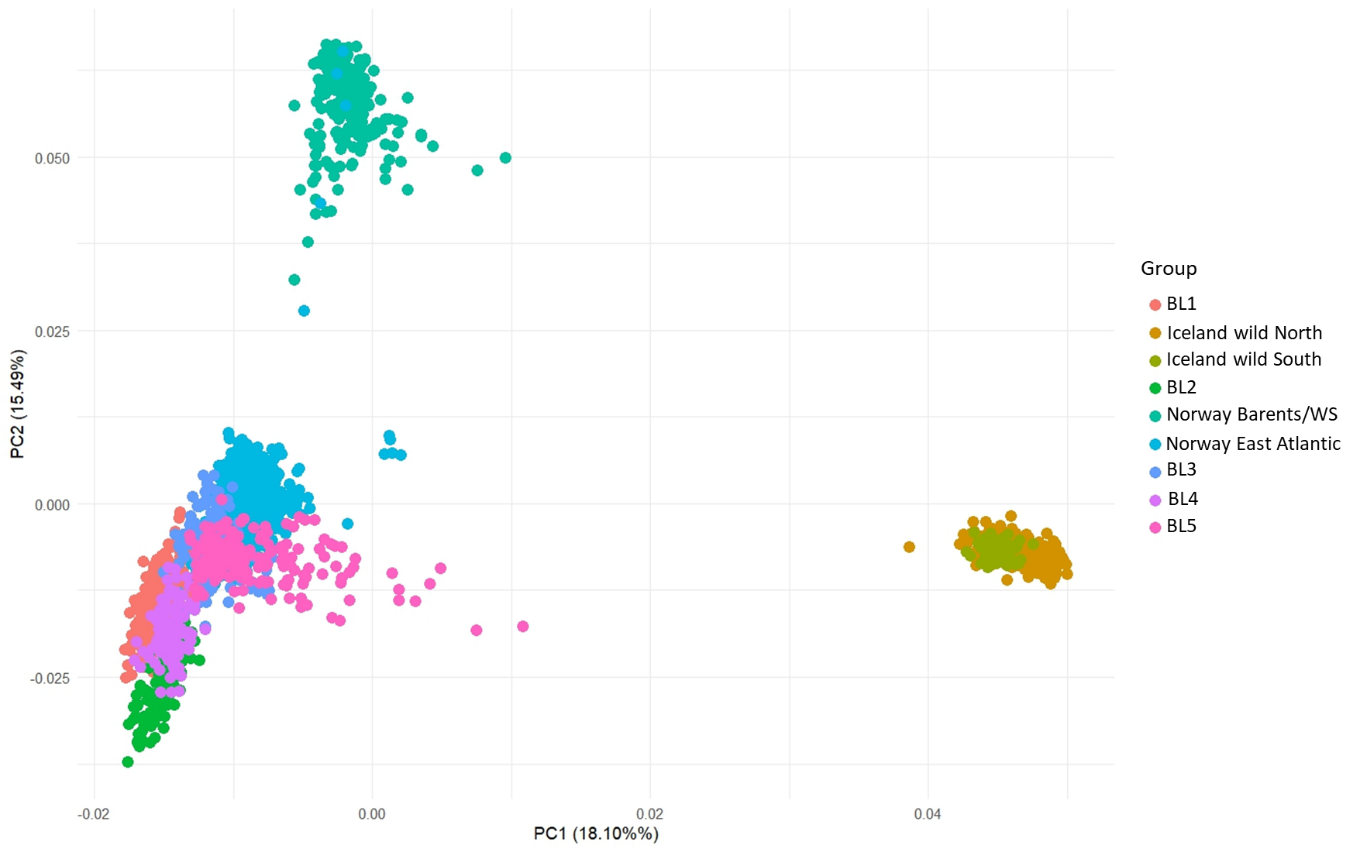


**Supplementary Figure 2:** Principal Component Analysis using 57 545 genome wide SNPs illustrating the genetic distance between the different phylogenetic groups in Norway and Iceland. The F_ST_ between the wild Norwegian Barents/White Sea and the wild Norwegian East Atlantic phylogenetic groups is 0.042. The F_ST_ between the wild Norwegian East Atlantic phylogenetic group and the pooled Icelandic group is 0.112. The F_ST_ between the wild Norwegian Barents/White Sea phylogenetic group and the pooled Icelandic group is 0.117.
